# Data on unstable charge/discharge behavior of composite anode composed of Sn compound and multi-walled carbon nanotube

**DOI:** 10.1016/j.dib.2018.02.023

**Published:** 2018-02-17

**Authors:** S.H. Kim, J.Y. Lee, Y.S. Yoon

**Affiliations:** **a** Department of Fashion Industry, Incheon National University Songdo, Yeonsu-gu, Incheon, Republic of Korea; b Department of Chemical Engineering, Gachon University, Gyeonggi-do 461-710, Republic of Korea

## Abstract

This data is related to the article entitled “Effect of Composite Structure on Capacity Instability of SnO_2_-Coated Multiwalled Carbon Nanotube Composite Anode” (Kim et al., 2018) [1]. This data provides the information about capacitance instability of a composite anode material based on multiwalled carbon nanotube (MWCNT) coated with crystalline and amorphous SnO_2_ and Sn on the inner and outer walls of MWCNT fabricated by a simple wet synthesis method.

**Specifications Table**

**Table:** 

Subject area	*Materials*
More specific subject area	*Composite Anode*
Type of data	*Table, figure*
How data was acquired	*BioLogic EC-Lab, VSP-300*
Data format	*Analyzed*
Experimental factors	*Half cell performance was measured with metal Li foil at RT*
Experimental features	*Coin cell performance*
Data source location	Energy Materials Lab, Department of Chemical Engineering, Gachon University, Republic of Korea.
Data accessibility	*This article*.

**Value of the data**

• Coin cell data on MWCNT-Sn compound composite anode.
• Data on instability of charge / discharge characteristics when Sn compound exists outside MWCNT
• Data on the optimum structure of anode composite composed of Sn compound and MWCNT.

## 1 Data

**Fig. 1:**
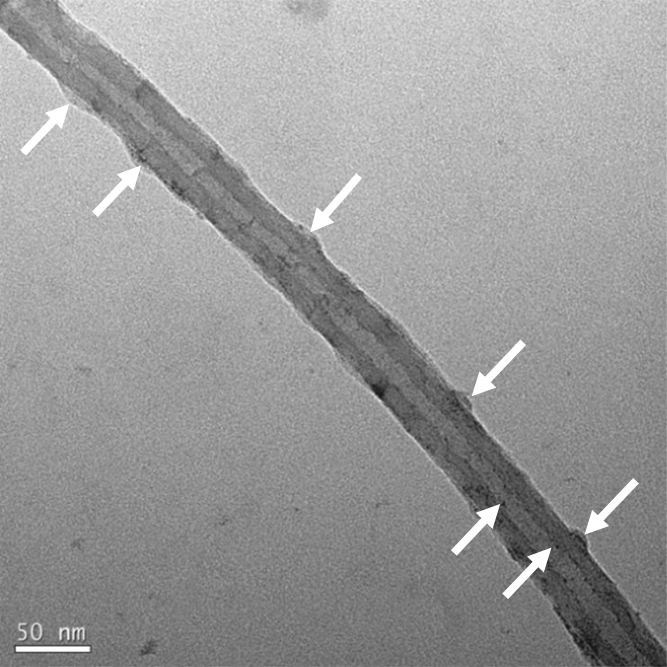
TEM image of a tin oxide coated MWCNT (white cursors show the tin oxide materials on outer and inner sides of MWCNT).

**Fig. 2:**
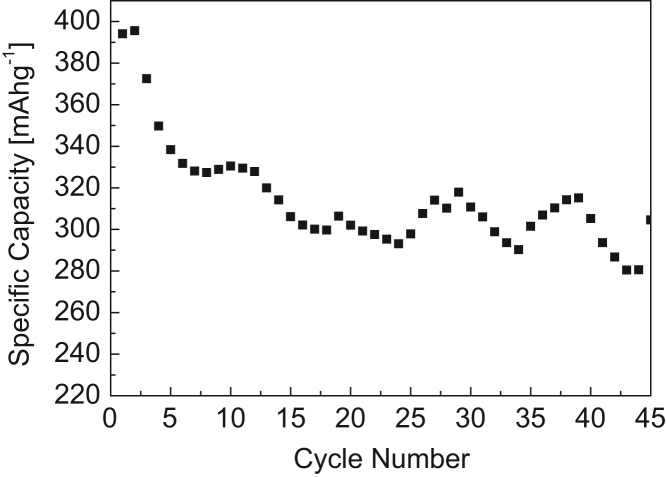
Capacities vs. cycle number for composite anode consisting of MWCNT- Sn compound.

This dataset provides information on the capacitance instability of MWCNT-Sn compound composite anode and the capacity variation characteristics of the composite with the progress of charge-discharge when the Sn compound is predominantly located on the outer surface of the MWCNT. Fig. 1 shows the TEM image of MWCNT-Sn based composite. Fig. 2 gives the graph for capacities vs. cycle number for MWCNT- Sn based composite anode materials obtained using the 2032 coin cell with metal Li. Table 1 shows representative values of the specific capacity according to the cycle number of the MWCNT-Sn compound composite anode synthesized at a precursor concentration of 0.5 M.

**Table 1:** Specific capacity according to the cycle number of the composite anode synthesized at a precursor concentration of 0.5 M.

Cycle Number	Specific capacity (mA h g^−1^)
20th	610
30th	670
40th	570

## 2 Experimental design, materials and methods

In order to chemically activate the surface of the MWCNT having an average diameter of 500 nm, a chemical surface treatment was carried out by the liquid phase method as follows. 1 g of MWCNT was stirred in 1 mol of nitric acid solution at 120 °C for 4 h and then washed with distilled water until pH 7. The resulting reaction product was then dried at 60 °C for 24 h. 0.105 g of SnC_2_O_4_·2H_2_O was mixed with 3 ml of distilled water and stirred at room temperature for 60 min. 3 ml of ethylene glycol and 0.25 g of poly-vinylpyrrolidone (PVP) were stirred at room temperature for 10 minutes. 0.33 g of the surface-treated MWCNT and thus obtained solution were mixed and heated to 195 °C and then stirred for another 5 h. The obtained reaction product was centrifuged, washed with distilled water and centrifuged again to obtain a precipitate. The precipitate was again dried in an electric oven at 50 °C for 5 h to obtain a composite anode in the form of a black powder.

A high resolution TEM analysis was performed to identify the shape and location of the hybridized Sn compounds in the MWCNT composite anode. An anode electrode slurry composed of 86 wt% of active material, 9 wt% of conductive material, and 5 wt% of binder was directly applied on the aluminum current collector to have a thickness of 50 μm and dried at 80 °C for 4 h. A CR2032 coin cell was prepared in a glove box using Li metal as the counter electrode and 1 M LiPF_6_ (EC: DMC: EMC=1: 1: 1) as the electrolyte. Specific capacities were measured using a cycling voltage and current method (CV) at a scan rate of 0.1 mV/s in the potential range of 0.1 and 2.5 V at room temperature (298 K).

## References

[1] Kim S.H., Lee J.Y., Yoon Y.S. (2018). Effect of composite structure on capacity instability of SnO_2_-coated multiwalled carbon nanotube composite anode. J. Alloy. Compd..

